# Supplementary material for: Multi‐dataset identification of innovative feature genes and molecular mechanisms in keratoconus
Source: J Cell Mol Med. 2024 Sep 19;28(18):e70079. doi: 10.1111/jcmm.70079 (PMC11412914; doi:10.1111/jcmm.70079)
Supplement: Supplementary file 2 — Material S2. [file JCMM-28-e70079-s002.zip › 7.GSEA analysis/4.MYRF/KEGG_ASCORBATE_AND_ALDARATE_METABOLISM.html]

Details for gene set KEGG\_ASCORBATE\_AND\_ALDARATE\_METABOLISM[GSEA]

|  || Dataset | MYRF.group.cls#h\_versus\_l |
| Phenotype | group.cls#h\_versus\_l |
| Upregulated in class | h |
| GeneSet | KEGG\_ASCORBATE\_AND\_ALDARATE\_METABOLISM |
| Enrichment Score (ES) | 0.30162218 |
| Normalized Enrichment Score (NES) | 0.8301124 |
| Nominal p-value | 0.73843056 |
| FDR q-value | 1.0 |
| FWER p-Value | 1.0 |
Table: GSEA Results Summary

  

Fig 1: Enrichment plot: KEGG\_ASCORBATE\_AND\_ALDARATE\_METABOLISM      
 Profile of the Running ES Score & Positions of GeneSet Members on the Rank Ordered List

  

| SYMBOL | TITLE | RANK IN GENE LIST | RANK METRIC SCORE | RUNNING ES | CORE ENRICHMENT || 1 | UGT1A8 | na | 369 | 0.620 | 0.2016 | Yes |
| 2 | UGT1A6 | na | 2556 | 0.320 | 0.2482 | Yes |
| 3 | UGT2B4 | na | 3822 | 0.260 | 0.3009 | Yes |
| 4 | UGT2B11 | na | 6521 | 0.178 | 0.2840 | Yes |
| 5 | UGT2B28 | na | 7657 | 0.147 | 0.3016 | Yes |
| 6 | ALDH3A2 | na | 11227 | 0.075 | 0.2244 | No |
| 7 | UGT2B10 | na | 12166 | 0.061 | 0.2182 | No |
| 8 | UGT2A3 | na | 16075 | 0.017 | 0.1115 | No |
| 9 | UGT2B7 | na | 16473 | 0.013 | 0.1045 | No |
| 10 | ALDH7A1 | na | 19743 | -0.019 | 0.0168 | No |
| 11 | UGDH | na | 19767 | -0.019 | 0.0226 | No |
| 12 | ALDH9A1 | na | 23662 | -0.070 | -0.0655 | No |
| 13 | ALDH1B1 | na | 25124 | -0.095 | -0.0750 | No |
| 14 | MIOX | na | 25177 | -0.096 | -0.0434 | No |
| 15 | UGT2B15 | na | 26025 | -0.113 | -0.0291 | No |
| 16 | UGT2A1 | na | 29490 | -0.205 | -0.0585 | No |
| 17 | ALDH2 | na | 34342 | -0.612 | 0.0111 | No |
Table: GSEA details [plain text format]

  

Fig 2: KEGG\_ASCORBATE\_AND\_ALDARATE\_METABOLISM      
 Blue-Pink O' Gram in the Space of the Analyzed GeneSet

  

Fig 3: KEGG\_ASCORBATE\_AND\_ALDARATE\_METABOLISM: Random ES distribution      
 Gene set null distribution of ES for **KEGG\_ASCORBATE\_AND\_ALDARATE\_METABOLISM**
